# Staff Conditions in Maternity Hospitals

**Published:** 1919-10-04

**Authors:** 


*To the Editor of* The Hospital.

### Staff Conditions in Maternity Hospitals.

*To the Editor of* The Hospital.

Sir,—"Seven pupil nurses" are to be congratulated on their courage in coming forward to right the wrong at Q.C.H. The Hospital would, indeed, be doing a good work in publishing the account of (1) Hours on and off duty. (2) Diet for staff. (3) Extra night work. (4) Accommodation for pupil midwives at maternity hospitals in this country.

In the maternity hospital where I obtained my C.M.B. the hours of work were 6.30 a.m. to 9 p.m. In addition to a large amount of responsible work, the trained pupils had to sweep floors, clean brass, wash up, etc. The off-duty every other day was 2 p.m. to 5 p.m., also one hour for study. On the alternate day we had 11 a.m. to 1 p.m. Very often this time was lost, but was *never* made up, and we were never given evening leave. Sundays we had four hours. As to accommodation: The pupils were housed away from hospital; miserable rooms, no attendance, except the weekly turn out and the shabbiest of furniture. The trained pupils usually had single rooms, but some of the untrained nurses were three in a room, day and night staff together. As to extra night work: The trained pupils got up for all operations and no extra leave given next day. The result of all this was that the pupils left worn out physically and mentally, and not fit to take up work without several weeks' rest, which they could ill afford. Thus in attempting to help one section of the community the hospital forgot that it was doing its best to ruin the health of another.

Yours faithfully, C.M.B.

*September* 27, 1919.

